# A survey of pathogens associated with *Cyperus esculentus L* (tiger nuts) tubers sold in a Ghanaian city

**DOI:** 10.1186/1756-0500-7-343

**Published:** 2014-06-06

**Authors:** Patrick F Ayeh-Kumi, Patience B Tetteh-Quarcoo, Kwabena O Duedu, Akua S Obeng, Kantanka Addo-Osafo, Samuel Mortu, Richard H Asmah

**Affiliations:** 1 Department of Microbiology, University of Ghana Medical School, Korle-Bu, Accra, Ghana; 2 Department of Medical Laboratory Sciences, University of Ghana School of Allied Health Sciences, Korle-Bu, Accra, Ghana

**Keywords:** Tiger nut, *Cyperus esculentus L*, Parasites, Bacteria, “atagwe” Milk, Ghana

## Abstract

**Background:**

*Cyperus esculentus L,* is a minor but important crop in Ghana. They are noted mostly by their aphrodisiac properties among others. The nuts are often eaten raw as an unprocessed snack due to its rich flavour and texture. Though eaten raw, the nuts are sometimes handled unhygienically, posing a public health threat. This study therefore aimed at determining the level and distribution of parasitic and bacterial contaminants associated with the crop as it is sold.

**Results:**

Four types of intestinal parasites were identified, and the most prevalent was *Cryptosporidium parvum* (30.0%). Nuts contaminated with parasites were found only among street vendors. Bacteriological examination showed three different groups of bacterial isolates with the most prevalent being coliforms (54.2%). Unlike parasites, bacteria isolates were found among samples from both street vendors and market places. Multiple drug resistance was displayed by *Proteus vulgaris*.

**Conclusions:**

Buying and eating nuts as well as other fruits taken raw from street vendors and market places could pose a significant public health threat. There is a need for efficient monitoring systems for food borne pathogens in Ghana.

## Background

**Figure 1:**
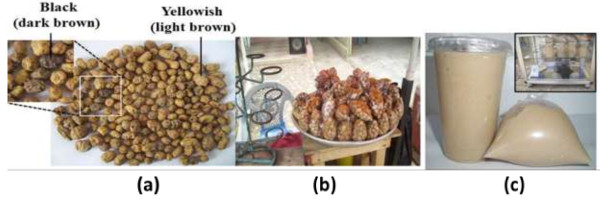
**Tiger nuts sold on the Ghanaian market. (a)** Types; the light brown or yellowish and the dark brown or blackish **(b)** Tiger nuts in plain polythene bags being sold at road side **(c)** Tiger nuts processed into porridge (locally known as ‘atagwe’ milk).

*Cyperus esculentus L*, also known as nutsedge, earth nuts, earth almond, rush nuts, and chufas, is a member of the grass family *Cyperaceae*[1]. The genus name *Cyperus* is from Cypeirus, an ancient Greek name, while the species name *esculentus* is of Latin origin meaning edible [2,3]. The nuts are a minor crop grown in the temperate and tropical zones of the world [4]. They have become naturalized in Ghana, Nigeria and Sierra Leone [5]. There are two types of tiger nuts (Figure 1a); the light brown type (or yellowish) and the black type (or dark brown) and they vary in size from about 1-3 cm in length to 1-2 cm in diameter [6].

Tiger nuts are consumed for their nutritional or medicinal values. Typically 100 g of the nuts contain 386 kcal (1635 kJ) of energy (7% of which are of proteins, 26% fats/oils, 31% starch and 21% glucose), and they contain an appreciable level of fibre (12% soluble and 14% non-soluble). They are also known to be rich in minerals such as sodium, calcium, iron, zinc, phosphorus, potassium, magnesium, copper and manganese [7]. In some parts of the world, they are medicinally known to have aphrodisiac, carminative, diuretic, emmenagogue and stimulating properties and also used in the preparation of tonics [8]. In Ayurvedic medicine, they are used for the treatment of flatulence, indigestion, colic, diarrhoea, dysentery, debility and excessive thirst [8,9].

In Ghana, the nuts are known and eaten mainly for their aphrodisiac property though the other uses are known to some people. They are sold packaged in plain polythene bags in markets and on the road side (Figure 1b). These nuts are sometimes processed into porridge (locally known as “atagwe” milk) and consumed domestically or sold to the public (Figure 1c). The majority of people buying the nuts purchases them whilst in a vehicle and consume without washing because they assumed sellers have washed already. Studies so far in some parts of the world have found some contaminants other than parasites. For example, in a small market survey of the beverage “Horchata” derived from tiger nuts frequently consumed in Southern Europe, aflatoxin B_1_ was detected [10]. In Nigeria, Adebanjo in 1993 found aflatoxins present at toxicologically unsafe levels on tiger nuts [11]. Besides aflatoxins, *Aspergillus flavus* have also been reported to contaminate tiger nuts [12]. Other contaminants such as stones, animal droppings and some other extraneous materials have been found to be associated with tiger nuts [13]. In most parts of Africa, the passion for eating outweighs other considerations such as food safety; this drive has led to considerable rise in food-borne intoxications and contamination [14]. The current study therefore, aimed at identifying common parasitic and bacterial contaminants on the crop, as it is sold and eaten by Ghanaians.

## Methods

### Study site & sample collection

The study was conducted at the Department of Microbiology, University of Ghana Medical School and the Department of Medical Laboratory Sciences, School of Allied Health Sciences, Korle-Bu, Accra (Ghana). Tiger nuts were bought at random from three popular markets and five traffic-prone streets in the Accra metropolis. At each site, 500 grams nuts were bought from five sellers. The market places were Agbogloshie, Kaneshie and Makola whereas the street vendors were located around UTC, Airport Residential, Shangrila, Madina and Kwame Nkrumah Circle. Prior to the collection of the samples, verbal interviews and brief discussions were held with the sellers to ascertain how they handled the nuts before packaging for sale.

### Ethical approval

The study protocol was approved by the Ethical and Protocol Review Committee of the School of Allied Health Sciences, University of Ghana. The aims and methods of the study were explained to the sellers who gave their consent before engaging them in the brief discussion on how the nuts were handled.

### Sample processing

Nuts from vendors at each site were put together and labelled by the name of the site, giving a total of eight (8) groups of about 500 g of nuts each. The nuts were examined macroscopically for the presence of worms and larval forms of insects. Each group of nuts was divided into five sub-groups (about 100 g each) for processing. The nuts were soaked in 150 ml of sterile normal saline, in sterile bottles for one hour. After the hour, the nuts were washed by shaking the bottles vigorously. The saline was then transferred into a sterile beaker. The process was repeated for three additional washes and all the washes pooled. Aliquots of the pooled saline washes were dispensed into centrifuge tubes and centrifuged at 5000 rpm for 5 minutes. The supernatant was decanted and the sediments were re-suspended in 1 ml of saline for parasitological examination while the other aliquot was used for bacteriological examination. The processes were repeated for each group of nuts collected. Sterile normal saline, in which washing of the nuts have not been done was also centrifuged, supernatant decanted, and used as control for both the parasitological and bacteriological examinations.

### Parasitological examination

Re-suspended sediments were examined by the direct wet mount and 10% formo-ether concentration techniques [15,16]. For the direct mount, briefly, a drop of sediment was placed on two clean, grease-free microscope slides. To one, a drop of iodine was added and both were covered with cover-slips. Each slide was then examined under a light microscope for the presence of parasite forms. Meanwhile, four slides each were prepared from the products obtained after the concentration process [15,16]. Two of the slides were examined like the direct wet mount with and without iodine. Following standard protocols [17], Trichrome and the modified Ziehl-Neelsen staining were performed on the other two slides. A drop of the centrifuged sterile saline was also treated and examined as described above, and that served as a control.

### Isolation, enumeration, identification and susceptibility testing of bacteria

Detection of bacteria and species identification were carried out using four (4) primary media (MacConkey, Sheep Blood Agar, Chocolate agar and Uriselect agar). The wash from each location, (part of which was used previously in the parasitological examination) was dispensed aseptically in 0.1 ml aliquots into separate petri dishes and pour plated with the primary media. As a control, equal volumes (0.1 ml) of sterile normal saline were also dispensed aseptically into petri dishes and pour plated with the media. The plates were kept for incubation at 37°C for 24 hrs. Bacterial colonies were counted on each plate after incubation to determine colony forming units (CFU). Bacteria isolates were further sub cultured to obtain pure cultures and then colonies were observed. Characterizations of isolates were made by microscopy, gram staining, morphologic examination, oxidation-fermentation tests and other biochemical tests including catalase test, urease test, triple sugar iron test, indole test and citrate utilization test [18-20].

Identified bacterial isolates were refined and their susceptibility patterns were determined for various antibiotics using a modified form of the Kirby Bauer method.

The antibiotics tested included gentamicin, amikacin, tetracycline, cotrimoxazole, cefotaxime, ceftizoxime, ampicillin, piperacillin, chloramphenicol, ciprofloxacin, levofloxacin and ofloxacin (Oxoid Ltd., Basingstoke, UK). These antibiotics are among the common antibiotics found on the Ghanaian market. The procedure for antibiotic susceptibility testing used is briefly described as follows. The test organism was emulsified in peptone water until the suspension became turbid and was comparable with 0.5% McFarland’s standard. A loopful of the suspension was transferred onto a Mueller-Hinton agar plate, after which a sterile cotton swab was used to streak the entire surface of the plate. Sterile forceps were used to apply the antibiotic discs to the surface of the agar plate and kept at 4°C for 4-6 hrs, so that the antibiotic can diffuse on the agar media. The plates were then incubated at 37°C for 18–24 hours. Zone diameters around the antibiotic discs were measured and later classified as sensitive or resistant based on the North Country Library System (NCLS) break point [21].

### Statistical analysis

The data collected were entered into MS Excel and analyzed using Minitab software version 15 (Minitab Inc. 2010). Descriptive analysis was carried out on the various microbial agents found on the tiger nuts, such as determination of their frequencies of occurrence and prevalence rates. Subsequently, Chi-square (χ2) was used to ascertain association between street vendors and market place contamination rates of the different microbial agents at p < 0.05.

## Results

Parasitological investigations found life form of four different parasites from the tiger nuts collected (Table 1). The parasites identified were in the following proportions; *Cryptosporidium parvum* oocysts (12/40, 30.0%), *Ancylostoma duodenale* ova (10/40, 25.0%), *Strongyloides stercoralis* larvae (9/40, 22.5%) and *Cyclospora cayetanensis* oocysts (9/40, 22.5%). *Cyclospora cayetanensis* was found to be present on nuts from three (3) of the five (5) street vendors studied, namely, UTC, Shangrila and Madina. *Cryptosporidium parvum*, the most predominant parasite found was present on nuts from two locations; UTC and Shangrila (Table 1). Interestingly, all parasites found were associated with nuts bought from street vendors (40/40, *P-Value* <0.001). Fifty percent (50% ) of the street vendors admitted washing their nuts in several changes of clean tap water before bagging for sale whereas 15% stated that they washed with salt water. The percentage of vendors that do not treat the nuts before selling was 25%. The remaining 10% could not tell how the nuts were treated before bagging because they buy already bagged nuts at a lower price from other vendors and sell to make profit. Meanwhile, there was no association between vendor washing the nuts and the number of parasites or bacterial colonies obtained (χ2 = 0.042, DF = 1, P-Value = 0.840).

**Table 1:** Parasites identified on tiger nuts from various locations in Accra

**Parasites**	Locations*				
	**UTC area**	**Shangrila area**	**Madina area**	**Airport area**	**Total no (%)**
*Strongyloides stercoralis* larvae	9	-	-	-	9 (22.5)
*Ancylostoma duodenale* ova	10	-	-	-	10 (25.0)
*Cryptosporidium parvum* oocysts	7	5	-	-	12 (30.0)
*Cyclospora cayetanensis*	4	3	2	-	9 (22.5)
**Total no (%)**	**30**	**8**	**2**	**0**	**40 (100)**

*No parasite was found in the tiger nuts bought from market places (Agbogbloshie, Kaneshie and Makola).

Overall, five bacterial species were isolated. Bacterial isolates were found to be associated with nuts bought from both street vendors and market place. The predominant isolates were grouped into coliforms, non-lactose fermenter (NLF) and *Staphylococcus* spp, and all showed varied colony forming units (Table 2). Different percentage of occurrences amongst all location was recorded. Among the coliforms were *Klebsiella oxytoca* (3/22, 13.6%), *Enterobacter cloacae* (7/22, 31.8%) *and Enterobacter spp* (3/22, 13.6%). NLF observed was *Proteus vulgaris* (1/22, 4.6%) whiles *Staphylococcus spp* recorded 36.4% (8/22).

**Table 2:** **Bacterial load of coliforms, non-lactose fermenter (NLF) and *Staphylococcus *** **spp (*Staph *** **spp.) among all locations**

**Location**	**Sample wt (g)**	**Coliforms (cfu/g)**	**NLF (cfu/g)**	***Staph *** **spp. (cfu/g)**	
**Samples from market places**					
Agbogloshie	100.2	1.2 × 10^4^	1.1 × 10^1^	3.0 × 10^3^	
Kaneshie	100.1	3.0 × 10^3^	2.0 × 10^1^	1.0 × 10^5^	
Makola	100.2	2.0 × 10^4^	0.0	3.2 × 10^4^	
**Samples from street vendors**					
Madina	100.0	2.2 × 10^4^	1.0 × 10^1^	2.2 × 10^2^	
Airport	99.8	3.0 × 10^1^	0.0	3.0 × 10^2^	
Shangrila	99.6	2.2 × 10^1^	0.0	3.0 × 10^1^	
Korle-Bu (UTC)	100.2	3.0 × 10^4^	0.0	2.0 × 10^4^	
Circle	99.8	2.0 × 10^5^	3.0 × 10^3^	3.0 × 10^1^	
*Sterile normal saline only	0.0	NC	NC	NC	

*Only sterile normal saline was cultured. NC means, no colonies observed and cfu/g means, colony forming unit per gram.

**Figure 2:**
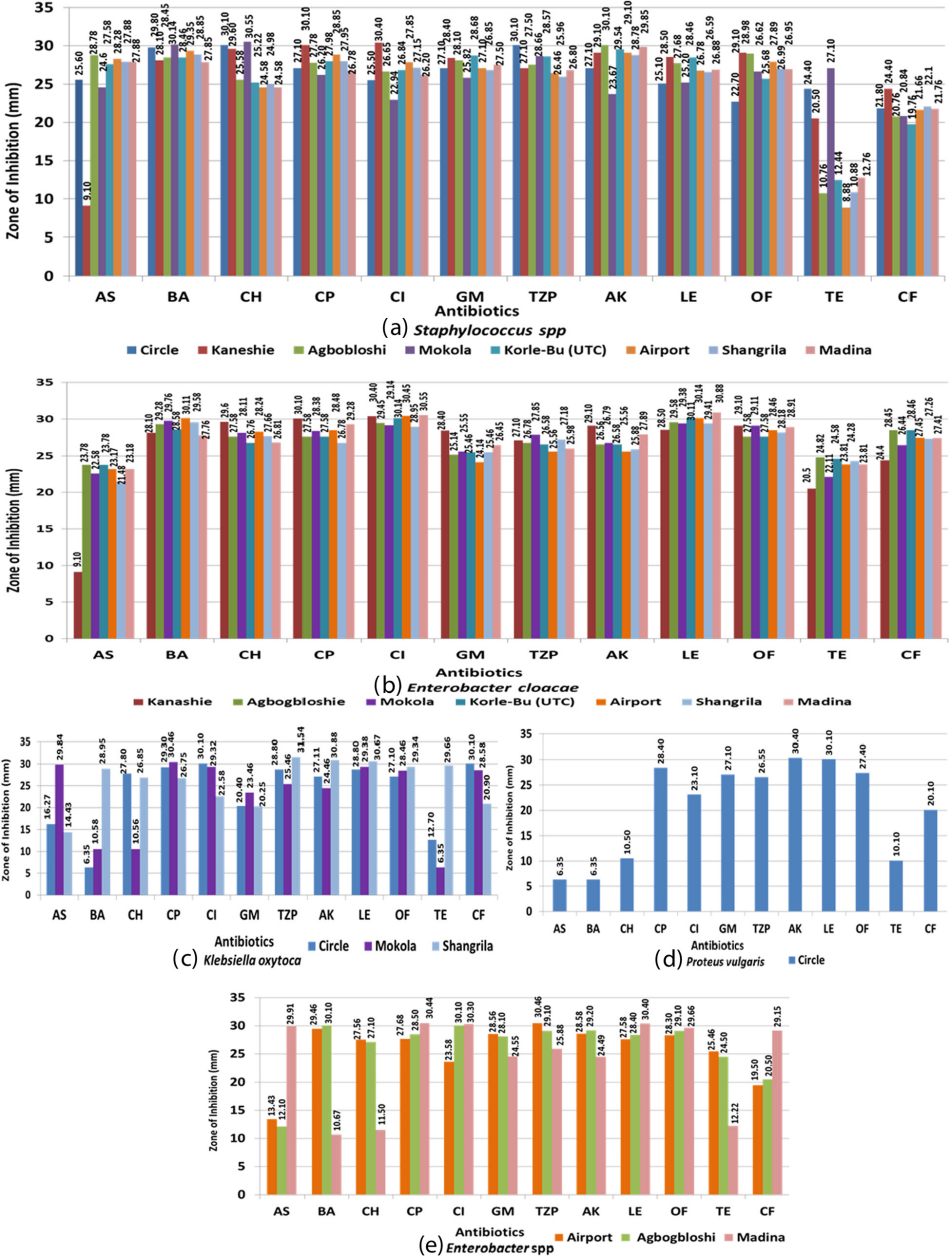
**Zone of inhibition recorded by bacteria isolated from tiger nuts bought from different locations. (a)***Staphylococcus* spp **(b)***Enterobacter cloacae***(c)***Klebsiella oxytoca***(d)***Proteus vulgaris***(e)***Enterobacter spp.* The antibiotics used, and the value of diameter measurement that were considered sensitive are: AS- ampicillin ≥15, BA- cotrimoxazole ≥16, CH- chloramphenicol ≥18, CP- ciprofloxacin ≥21, CI-ceftizoxime ≥ 25, GM-gentamicin ≥ 15, TZP- piperacillin ≥21, AK-amikacin ≥ 17, LE-levofloxacin ≥ 17, OF-ofloxacin ≥ 16, TE-tetracycline ≥ 15, CF- cefotaxime ≥26. Numbers on each bar indicate the exact measurement of the inhibition zone in mm.

Some locations recorded more isolates than others (Figure 2). Inhibition zones have been presented in Figure 2, showing the isolates that were sensitive or resistance to different abtibiotics. These included the resistance of *Staphylococcus* spp from Kaneshie to ampicillin and resistance of *Staphylococcus* spp from Airport, Agbogloshie, Makola and Madina, to tetracycline (Figure 2a). *Enterobacter* spp from Agbogbloshi and Airport, and *Klebsiella oxytoca* from Shangrila were also resistant to ampicillin. Also, *Enterobacte*r spp from Airport and Madina, and *K. oxytoca* from Mokola were all resistant to cotrimoxazole*. Enterobacter spp* from Madina and *K. oxytoca* from Mokola were resistant to chloramphenicol*. K. oxytoca* from Mokola and Circle, and *Enterobacter spp* from Madina were all resistant to tetracycline (Figure 2b). *Enterobacter Cloacae* from Kaneshie was resistant to ampicillin. One notable pattern was the multiple resistance of *Proteus vulgaris* from Circle to ampicillin, cotrimoxazole, chloramphenicol and tetracycline (Figure 2c).

Antibiotic sensitivity patterns of the bacteria isolated from the tiger nuts are reported in Table 3. In the present study *Staphylococcus* spp was highly sensitive to all the antibiotics tested, except Tetracycline. *K. oxytoca* was highly sensitive to ciprofloxacin, gentamicin, piperacillin, amikacin, levofloxacin and ofloxacin, relatively sensitive to cefotaxime and ampicillin, but resistant to cotrimoxazole and tetracycline. *P. vulgaris* was highly sensitive to 7 of the antibiotics (ciprofloxacin, ceftizoxime, piperacillin, amikacin, levofloxacin, ofloxacin and gentamicin), but was resistant to 5 antibiotics, including ampicillin, cotrimoxazole, chloramphenicol, tetracycline and cefotaxime. *Enterobacter spp.* displayed sensitivity to all antibiotics with the exception of ampicillin and cefotaxime. *E. cloacae* showed sensitivity to all the antibiotics.

**Table 3:** Antibiotic sensitivity pattern of bacteria isolated from tiger nuts

**Antibiotics**			**Bacteria Isolates**		
	***Staphylococcus *** **spp. (n = 8)**	***E. cloacae *** **(n = 7)**	***K. oxytoca *** **(n = 3)**	***P. vulgaris *** **(n = 1)**	***Enterobacter spp. *** **(n = 3)**
Ampicillin	87.5	85.7	66.67	0	33.3
Cotrimoxazole	100	100	33.3	0	66.7
Chloramphenicol	100	100	66.67	0	66.7
Ciprofloxacin	100	100	100	100	100.0
Ceftizoxime	87.5	100	66.67	100	66.7.0
Gentamicin	100	100	100	100	100.0
Piperacillin	100	100	100	100	100.0
Amikacin	100	100	100	100	100.0
Levofloxacin	100	100	100	100	100.0
Ofloxacin	100	100	100	100	100.0
Tetracycline	37.5	100	33.3	0	66.7
Cefotaxime	87.5	85.7	66.67	0	33.3

n = number of bacteria isolates. Total number of location = 8.

## Discussion

In this study, we investigated bacterial and parasitic contamination of *Cyperus esculentus L* sold on the Ghanaian market and street. A wide range of both bacterial and parasitic contaminants were detected. A number of factors, such as direct contact of nuts with the soil, unhygienic handling, and the use of contaminated water for irrigation, could have accounted for the contamination observed. These factors make the nuts prone to parasitic and bacterial agents that have long been associated with fresh vegetables and fruits [22-26]. An indirect factor that could have also contributed to the contamination observed in the current study is urbanization. The increase in urbanization [27-29] has resulted in high food demand and a similar pressure on social amenities like clean water. Access to clean potable water is a challenge in the Accra metropolis. As a result, vendors who require larger amounts of water to wash food items do so, not under running water, but in a bowl. This practice is likely to have resulted in the contaminants found on the nuts.

Five bacterial agents, some with serious health implications, were found in the current investigation. Studies have shown that individuals with recurrent infections and those with structural abnormalities of the urinary tract have an increased frequency of infection caused by *Proteus vulgaris* and other bacteria such as *Klebsiella* spp, Enterobacter, Pseudomonas, enterococci and staphylococci [30,31]. Hence isolation of some of these bacteria on the tiger nuts is a health concern. Although *P. vulgaris* recorded low prevalence rate (4.6%), its presence is reliable proof of faecal contamination of the tiger nuts which could be as a result of irrigation with contaminated water sources and direct soil contact. This practice is common in developing countries, with Ghana not being an exception. *P. vulgaris* and *Staphylococcus* spp. have been implicated in food spoilage and food-borne diseases, hence, they should be of interest when food hygiene is being considered [22]. This supports the assertion that, the intake of contaminated food can cause diarrhoeal-associated illnesses with bacteria being one of the major causes [32]. Yeboah-Manu *et al*., reported high levels of bacterial contamination among sellers in Accra, which included contaminants such as *Staphylococcus* spp*., Enterobacter cloacae* and *Proteus spp*[33], therefore, the hygiene and packaging methods of these tiger nut must be monitored. Bacterial counts on tiger nuts showed different bacterial loads. All the samples bought from market places recorded unacceptable levels of coliforms, which are indicators of food hygiene. This result is expected since the market women do not seem to wash the nuts before selling it to the vendors. Although most of the vendors interviewed (including those from Korle-Bu and Circle) admitted they washed their products with several changes of tap water before bagging and selling, this level of contamination is not surprising because, chlorinated water in distribution here in Accra has been found to be contaminated [34]. Even sachet water, which is regarded as being more purified, has been identified to contain bacteria counts ranging from 2.8 × 10^3^ to 5.9 × 10^5^ cfu/mL [34]. The high microbial loads of the tiger nut sampled from the study locations, which were unacceptable, supports the finding of Nyarko *et al*. [35]. In that study, they found high levels of microbial loads on non-sterilized tiger nuts [35]. Vendors from Shangrila and Airport residential area recorded low bacterial levels. This could be because, the aforementioned locations are regarded as residence for the elite class in the city. The vendors, therefore, wash the nuts to try and make them presentable, and in the process reduce bacterial contaminants, before bagging.

From the outcome of the sensitivity testing, it is noteworthy that, *Staphylococcus* spp, *K. oxytoca* and *P. vulgaris* were all resistant to tetracycline. Two out of these bacteria (*K. oxytoca* and *P. vulgaris*), together with *Enterobacter spp.*, were resistant to ampicillin whiles *K. oxytoca* and *P. vulgaris* were resistant to cotrimoxazole (Table 3). *Proteus vulgaris* showed multiple resistance to ampicillin, cotrimoxazole, chloramphenicol and tetracycline. These antibiotics, especially tetracycline and cotrimoxazole, have been on the Ghanaian market for a long time and therefore might have been exposed to frequent use or abuse. This might account for the levels of resistance observed. The resistance recorded in the current study supports the finding of other researchers who also worked on isolates from food items [36,37]. These researchers therefore suggested that the incidence of resistant bacteria (especially enterobacterial pathogens) is a worldwide phenomenon and a major public health threat [36,37]. All the bacterial isolates were sensitive to Ciprofloxacin, Piperacillin, Gentamicin, Amikacin, Levofloxacin and Ofloxacin. This finding suggests that, medical officers can have a numbers of options to choose from, when treating bacterial infection acquired from the consumption of contaminated tiger nuts sold on the Ghanaian market.

The parasitic contaminants found on the nuts are associated with gastrointestinal infections [38]. To the best of our knowledge, this is the first study to report parasitic contamination of tiger nuts sold in a Ghanaian city. The most prevalent parasite, *Cryptosporidium parvum* (30.0%), is known to contaminate both food and water, and have sometimes caused small and large outbreak of acute watery diarrhoea [39,40]. Occurrence of this parasite on the nuts supports the suggestion that contaminated leafy vegetables and fruits can be connected to Cryptosporidium *parvum* transmission [41,42]. Although *Cryptosporidium parvum* is regarded as a minimally invasive mucosal pathogen, infection can be associated with diarrhoea and marked mucosal inflammation [43]. Some cases of pulmonary and tracheal cryptosporidiosis, coughing and fever can also be seen [44]. Mostly, the diarrhoea is self-limiting; however, immuno-compromised individuals can develop uncontrollable forms leading to severe dehydration that is potentially fatal [45]. Parasites such as *Ancylostoma duodenale* and *Strongyloides stercoralis* are common species of soil-transmitted helminthes, which can be related to infection associated with tiger nut consumption considering the way it is cultivated. Infection of these parasitic agents can cause significant nutritional deficiencies, delayed physical and cognitive development during childhood, and reduced productivity in adults [46,47]. In spite of *Cyclospora cayetanensis* recording low prevalence (22.5%), its occurrence should not be overlooked, since it has been recognized as an emerging food and water-borne pathogen that causes protracted diarrhoea in humans [48]. The clinical presentation is somewhat different in areas with varying levels of endemicity. Nevertheless, younger children have more severe clinical symptoms [48]. Coinfection of *Cyclospora cayetanensis* with *Cryptosporidium parvum* and other parasites has been described for both immunocompetent and immunocompromised individuals to cause more severe presentations [49], and this is noteworthy since both parasites were found to be present on the tiger nuts. In spite of the good nutritional and medicinal values of tiger nuts, consumption of untreated nuts has the potential of increasing transmission of both intestinal parasites and pathogenic bacteria that could lead to a rise in water and food-borne infections if not properly processed. Washing with salt water promises to be an effective decontamination procedure. Another method that is also recommended is surface sterilization. This method of decontamination is in conformity with the study conducted by Yousuf *et al.,* in which different chemicals including calcium hypochlorite solution and oxalic acid in a form of lemon juice were used, as decontaminants of shrimp and prawn, and yielded significant reduction in bacterial contamination [50]. In view of this, investigation on agricultural practices of farmers and vendors that tend to expose the nuts to contaminants need to be undertaken periodically to monitor transmission of bacterial and parasitic diseases.

## Conclusions

Buying and eating fruits and vegetables from street vendors without further washing could result in infection with pathogenic parasites and bacteria. Pre-treated fruits or vegetables being sold on both streets and markets in Ghana could be a potential source of transmission of food borne diseases. There is therefore a need for regular monitoring of the quality of such food items sold. There is also the need for consumers to pay critical attention to proper washing and surface sterilization of the tiger nuts before consumption.

## Abbreviations

NLF: Non-lactose fermenter; CFU: Colony-forming unit; K. oxytoca: *Klebsiella oxytoca*; E. cloacae: *Enterobacter cloacae*; Enterobacter spp: *Enterobacter* specie; P. vulgaris: *Proteus vulgaris*; Staphylococcus spp: *Staphylococcus* species; C. parvum: *Cryptosporidium parvum*; NCLS: North Country Library System; hrs: hours; mm: millimeters.

## Competing interests

The authors declare that they have no competing interests.

## Authors’ contributions

PFA-K conceived and designed the experiments: KOD participated in collection and analyses of the data and drafting of the manuscript. ASO contributed in interpretation of the data. PBT-Q and RHA Jointly developed the structure and arguments for the manuscript. SM and KA-O made critical revisions and approved final version. All authors read and approved the final manuscript.

## References

[B1] Cobley SL Introduction to the botany of tropical crops 1962 London: Longman

[B2] Okladnikov Iu N Vorkel' Ia B Trubachev IN Vlasova NV Kalacheva GS Inclusion of chufa in the human diet as a source of polyunsaturated fatty acids Vopr Pitan 1977 3 45 48 883233

[B3] Mohamed LS Mohsen Z Imaizumi K Dietary supplementation with Cyperus esculentus L (tiger nut) tubers attenuated atherosclerotic lesion in apolipoprotein E knockout mouse associated with inhibition of inflammatory cell responses Am J Immunol 2005 1 1 60 67

[B4] Tetteh JP Offorin E A baseline survey of tiger nut (Cyperus esculentus) production in the Kwahu South District of Ghana Ghana J Agric Sci 1998 31 2 211 216

[B5] Cyperules AA The new Encyclopedia Britannica 1992 15 Chicago: Macropaedia p. 185

[B6] Maud JK Processing and Preservation of Tropical and Subtropical Foods 1991 Hong Kong: MacMillan Education Ltd

[B7] Sanful RE The use of tiger-nut (Cyperus esculentus), cow milk and their composite as substrates for yoghurt production Pak J Nutr 2009 6 755 758

[B8] Chevallier A The encyclopedia of medicinal plants 2001 Australia: Dorling Kindersley

[B9] Chopra RN Glossary of Indian medicinal plants 1992 New Delhi: Publication and Information Directorate, CSIR

[B10] Arranz I Stroka J Neugebauer M Determination of aflatoxin B1 in tiger nut-based soft drinks Food Addit Contam 2006 23 3 305 308 10.1080/02652030500415652 16517532

[B11] Adebajo LO Survey of aflatoxins and ochratoxin A in stored tubers of Cyperus esculentus L Mycopathologia 1993 124 1 41 46 10.1007/BF01103055 8159216

[B12] Bankole SA Adebanjo A Mycotoxins in food in West Africa: current situation and possibilities of controlling it Afr J Biotechnol 2003 2 9 254 263

[B13] Mordi JI Okafor JNC Ozumba AU Solomon HM Olatunji O Contaminants and defects in Nigerian Tiger-nut varieties J Trop Sci 2006 46 3 141 142 10.1002/ts.71

[B14] Miller JD Cardwell KF Mycotoxins Proceedings of the workshop on mycotoxins in food in Africa 1996 Cotonou, Benin: International Institute of Tropical Agriculture, Benin 18 22

[B15] Cheesbrough M District laboratory practice in tropical countries. 2nd ed. Cambridge 2005 New York: Cambridge University Press

[B16] Schwartzbrod J Methods of analysis of helminth eggs and cysts in wastewater, sludge, soils and crops 1998 Nancy, France: University Henry Poincare

[B17] CDC Staining procedures. Atlanta, Georgia: Centers for Disease Control and Prevention http://www.cdc.gov/dpdx/diagnosticProcedures/stool/staining.html. [Accessed July 28, 2011]

[B18] Harrigan WF McCance M E. Laboratory Methods in Food Diary Microbiology 1993 London, UK: Academic Press

[B19] Buchanan RE Gibbon NE Bergeys Manual of Determinative Bacteriology 1974 Baltimore, USA: Williams and Wilkins Co

[B20] Abbey SD Foundation in Medical Mycology 2007 4 Port Harcourt, Nigeria: Kenalf Publication 22 30

[B21] Barry AL Jones RN Criteria for disk susceptibility tests and quality control guidelines for the cefoperazone-sulbactam combination J Clin Microbiol 1988 26 1 13 17 PMCID: PMC266166 3343304 10.1128/jcm.26.1.13-17.1988 PMC266166

[B22] Nester EW Anderson DG Roberts CE Nancy J Pearsall NN Nester MT Microbiology: A Human Perspective 2004 4 London: McGraw Hill 801 814

[B23] Choi DW Lee S Incidence of parasites found on vegetables collected from markets and vegetable gardens in Taegu area Kisaengchunghak Chapchi 1972 10 1 44 51 12913510 10.3347/kjp.1972.10.1.44

[B24] Choi WY Chang K The incidence of parasites found of vegetables Kisaengchunghak Chapchi 1967 5 3 153 158 12913557 10.3347/kjp.1967.5.3.153

[B25] Lee JS Seo JS Ock MS Park YS Change in incidence of parasite eggs and larvae from vegetables in the markets of Taegu Kisaengchunghak Chapchi 1983 21 1 105 110 12902676 10.3347/kjp.1983.21.1.105

[B26] Iwanczuk I Examination of vegetables and fruits in Warsaw markets for the presence of eggs of human intestinal parasites Przegl Epidemiol 1963 17 223 230 14061468

[B27] World Bank Bank W World development report 2000/2001 Attacking poverty 2000 Washington, DC: Oxford University Press

[B28] Ghana Statistical Service 2000 population and housing census: Summary report of final results 2002 Accra: The GSS

[B29] Ghana Statistical Service Population and Housing census: special report on 20 largest localities 2002 Accra: The GSS

[B30] Ronald A The etiology of urinary tract infection: traditional and emerging pathogens Am J Med 2002 113 Suppl 1 14 19 10.1016/s0002-9343(02)01055-0 12113867

[B31] Fluit AC Jones ME Schmitz FJ Acar J Gupta R Verhoef J Antimicrobial resistance among urinary tract infection (UTI) isolates in Europe: results from the SENTRY Antimicrobial Surveillance Program 1997 Antonie Van Leeuwenhoek 2000 77 2 147 152 10.1023/A:1002003123629 10768473

[B32] Mensah P Mwamakamba L Mohamed C Nsue-Milang D Public Health and Food Safety in the WHO African region Afr J Food Agric Nutr Dev 2012 12 4 6317 6335

[B33] Yeboah-Manu D Kpeli G Akyeh M Bimi L Bacteriological quality of ready-to-sell foods sold in and around University of Ghana campus Res J Microbiol 2010 5 130 136

[B34] Tagoe DNA Nyarko H Arthur SA Birikorang E A study of antibiotic susceptibility pattern of bacteria isolates in sachet drinking water sold in the cape coast metropolis of Ghana Res J Microbiol 2011 6 453 458

[B35] Nyarko HD Daniel N Tagoe A Aniweh Y Assessment of microbiological safety of tiger nuts (Cyperus esculentus L.) in the Cape Coast Metropolis of Ghana Arch Appl Sci Res 2011 3 6 257 262 ISSN 0975-508X. CODEN (USA) AASRC9

[B36] Rahman K Malik A Antibiotic resistance and detection of β - lactamase in bacterial strains of Staphylococci and Escherichia coli isolated from foodstuffs World J Microb Biotechnol 2001 17 863 10.1023/A:1013857101177

[B37] Vyas P Antibiotic resistance pattern of mdr pathogens IOSR J Pharm 2012 2 5 44 46

[B38] World Health Organization Informal Consultation on Intestinal Parasite Infections 1996 Geneva, Switzerland: World Health Organization WHO Document No. WHO/CDC/PI/90.1

[B39] Centers for Disease Control and Prevention Cryptosporidiosis—New Mexico, 1986 Morb Mortal Wkly Rep 1987 36 561 563 3112550

[B40] Mac Kenzie WR Hoxie NJ Proctor ME M Gradus MS Blair KA Peterson DE Kazmierczak JJ Addiss DG Fox KR Rose JB Davis JP A massive outbreak in Milwaukee of Cryptosporidium infection transmitted through the public water supply N Engl J Med 1994 331 3 161 167 10.1056/NEJM199407213310304 7818640

[B41] Laberge I Griffiths MW Griffiths MW Prevalence, detection and control of Cryptosporidium parvum in food Int J Food Microbiol 1996 32 1 26 10.1016/0168-1605(96)00977-4 8880324

[B42] Robertson LJ Gjerde B Occurrence of parasites on fruits and vegetables in Norway J Food Prot 2001 64 1793 1798 11726161 10.4315/0362-028x-64.11.1793

[B43] Laurent F McCole D Eckmann L Kagnoff MF Pathogenesis of Cryptosporidium parvum infection Microbes Infect 1999 1 2 141 148 10.1016/S1286-4579(99)80005-7 10594978

[B44] Abrahamsen M Templeton T Enomoto S Abrahante J Zhu G Lancto C Deng M Liu C Widmer G Tzipori S Buck G Xu P Bankier A Dear P Konfortov B Spriggs H Lyer L Anantharaman V Aravind L Kapur V Complete genome sequence of the apicomplexan, cryptosporidium parvum Science 2004 304 441 445 10.1126/science.1094786 15044751

[B45] Foodborne Pathogenic Microorganisms and Natural Toxins Handbook, Cryptosporidium parvum. U.S. Food and Drug Administration http://www.fda.gov/downloads/Food/FoodborneIllnessContaminants/UCM297627.pdf

[B46] Stephenson LS Helminth parasites, a major factor in malnutrition World Health Forum 1994 15 169 172 8018283

[B47] Santos FLN Souza AMGC Soares NM Hookworm and threadworm infections and their association with hemoglobin and eosinophil concentrations in residents of Salvador-Bania, Brazil Rev Inst Med Trop 2013 55 4 233 238 doi:10.1590/S0036-46652013000400003 10.1590/S0036-46652013000400003

[B48] Ynés RO Sanchez R Update on cyclospora cayetanensis, a food-borne and waterborne parasite Clin Microbiol Rev 2010 23 1 218 234 doi:10.1128/CMR.00026-09 10.1128/CMR.00026-09 20065331 PMC2806662

[B49] Bellagra N Ajana F Coignard C Caillaux M Mouton Y Co-infection with cryptosporidium sp. and cyclospora sp. in an AIDS stage HIV patient Ann Biol Clin 1998 56 476 478 9754285

[B50] Yousuf AHM Ahmed MK Yeasmin S Ahsan N Rahrman MM Islan MM Reduction of bacterial pathogens in penaeus monodon and macrobrachium rosenbergii using several chemical interventions World J Agric Sci 2008 4 Suppl 856 861

